# Supplementary material for: Structural basis for the transport and regulation mechanism of the multidrug resistance-associated protein 2
Source: Nat Commun. 2025 Jan 8;16:484. doi: 10.1038/s41467-024-55810-w (PMC11711199; doi:10.1038/s41467-024-55810-w)
Supplement: Supplementary file 1 — Supplementary Information [file 41467_2024_55810_MOESM1_ESM.pdf]

Supplementary Information

Structural basis for the transport and regulation mechanism of the Multidrug resistance-associated protein 2

Eriko Koide[1], Harlan L. Pietz[1,2], Jean Beltran[3], and Jue Chen[1,4,*]

[1]Laboratory of Membrane Biology and Biophysics, The Rockefeller University, New York, NY 10065, USA.

[2]Weill Cornell/Rockefeller/Sloan Kettering Tri-Institutional MD-PhD Program, New York, NY 10065, USA.

[3]Department of Biology, Davidson College, Davidson, NC 28035, USA

[4]Howard Hughes Medical Institute, The Rockefeller University, 1230 York Ave, New York, NY 10065, USA.

*To whom correspondence should be addressed: juechen@rockefeller.edu.

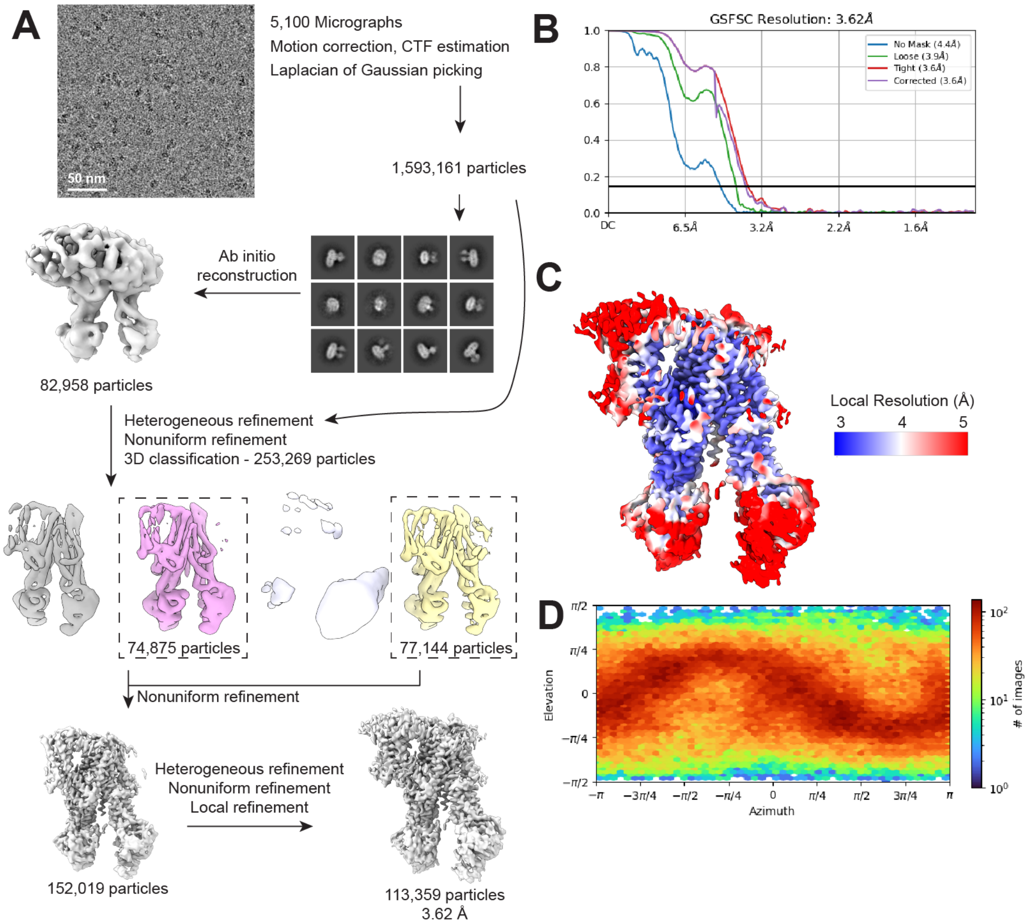

Supplementary Figure 1. Structure determination of the ligand-free MRP2.

(A) The image processing workflow.

(B) Fourier shell correlation (FSC) curves of the final map generated in CryoSPARC. Blue: no mask applied; green: with a soft solvent mask: red: with a tight mask around the protein; and purple: “Corrected” represents FSC calculated after applying the tight mask and correcting by noise substitution[1].

(C) Local resolution estimation of the final reconstruction.

(D) Euler angle particle distribution plot.

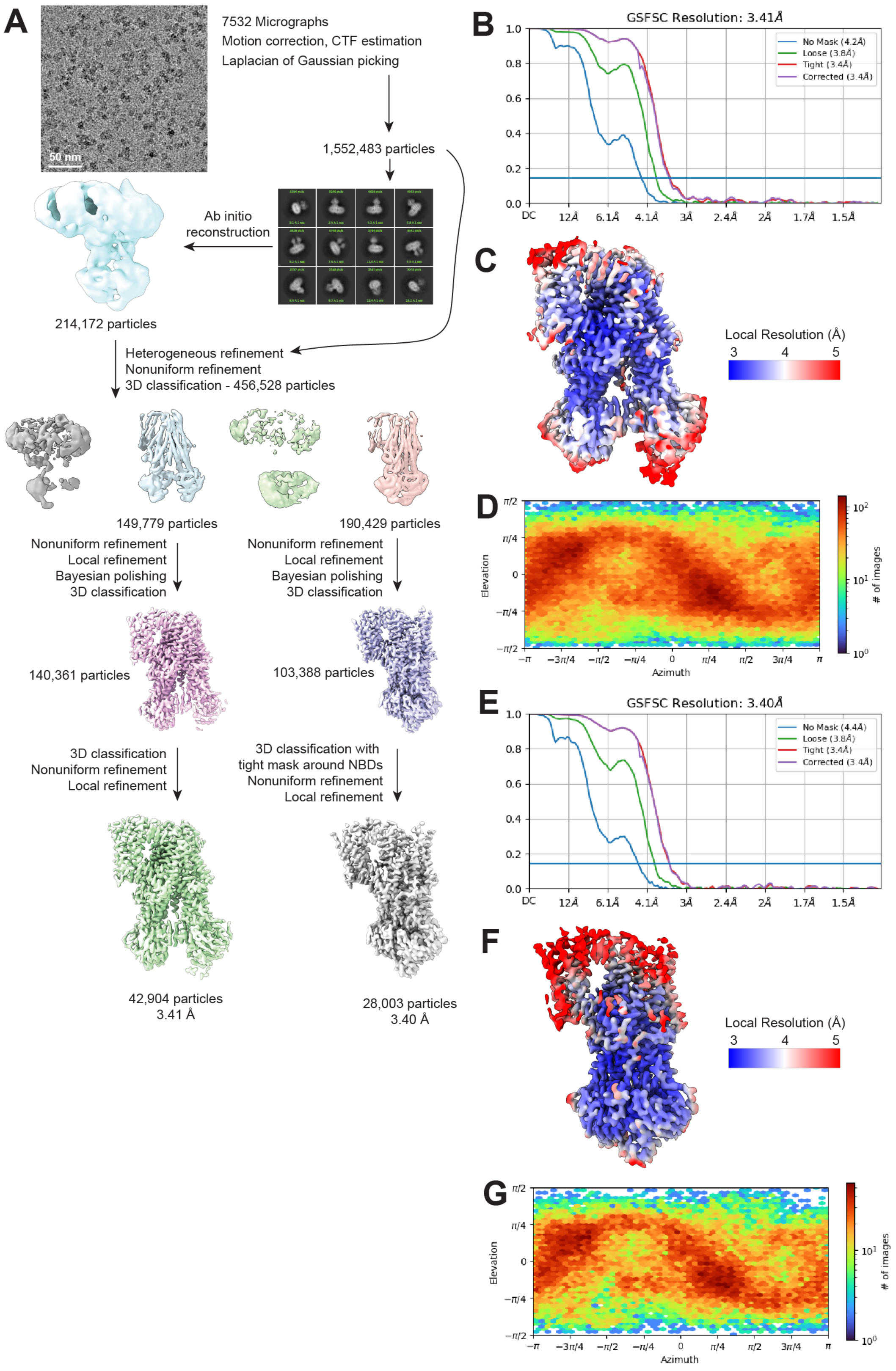

Supplementary Figure 2. Structure determination of the E1462Q variant in the presence of ATP.
(A) Image processing workflow.
(B) Fourier shell correlation curves of the inward-facing conformation.
(C) Local resolution estimation for the inward-facing conformation.
(D) Euler angle particle distribution plot for the inward-facing conformation.
(E) Fourier shell correlation curves of the NBD-dimerized conformation.
(F) Local resolution estimation for the NBD-dimerized conformation.
(G) Euler angle particle distribution plot for the NBD-dimerized conformation.

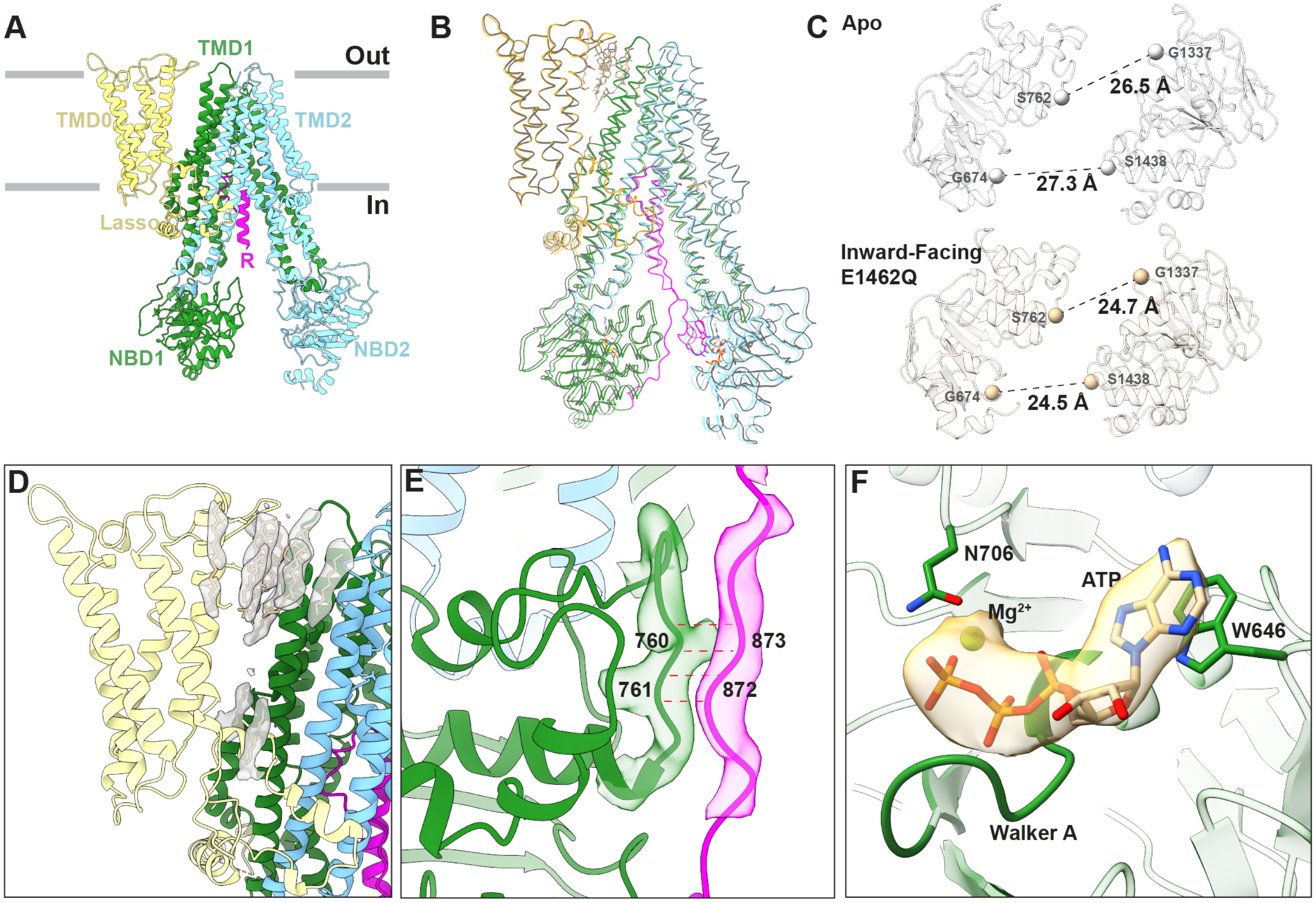

Supplemental Figure 3. Two similar auto-inhibited structures

(A) The overall structure of the WT MRP2 in the ligand-free, apo conformation.

(B) Structural superposition of the E1462Q inward-facing conformation (colored) and the WT apo conformation (gray).

(C) Structural comparison of the NBDs of observed in the WT apo and E1462Q inward-facing conformations. Distances between pairs of ATP binding residues are indicated.

(D) Structure of the TMD0 observed in the MRP2(E1462Q) reconstruction, with cryo-EM density at contour level of 0.0596 corresponding to cholesterol and lipid depicted as surface.

(E) Residues 758-762 of NBD1 closely interact with the R domain as it extends from NBD1, forming a beta sheet with residues 760 and 761. Cryo-EM density (contour level 0.0792) at site of interaction is depicted as transparent surface.

(F) ATP bound to NBD1 observed in the MRP2(E1462Q) inward-facing structure. Cryo-EM density (contour level 0.0941) for ATP and magnesium are shown as yellow surface.

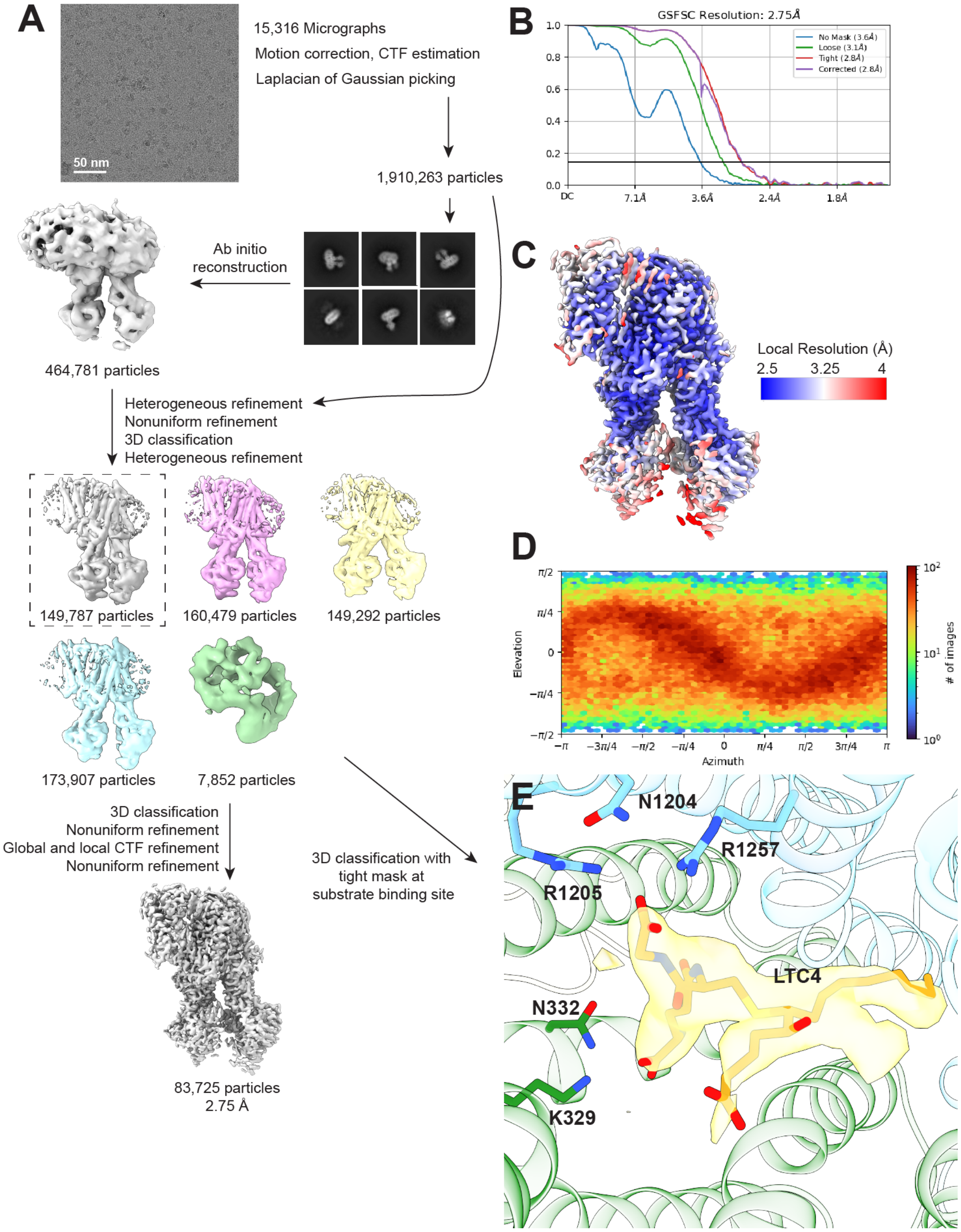

Supplementary Figure 4. Structure determination of the LTC4-bound structure.

(A) Image processing workflow.

(B) Fourier shell correlation curves.

(C) Local resolution estimation.

(D) Euler angle particle distribution.

(E) Cryo-EM density (yellow) corresponding to LTC4 at contour level 0.00174 in the substrate-binding site. The hydrophobic tail of LTC4 is not modeled as clear corresponding density was not observed in this region.

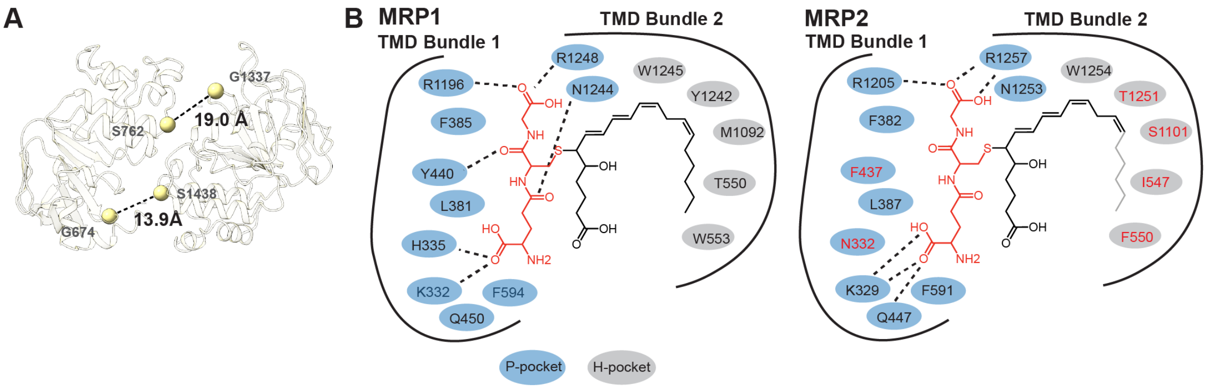

Supplemental figure 5. The LTC4-bound conformation

(A) The structure of the NBDs observed in the LTC4-bound MRP2 reconstruction.

(B) Schematic drawings comparing LTC4-binding in MRP1 and MRP2**.** The polar P-pocket residues of MRP1 and their corresponding residues in MRP2 are depicted in blue, and hydrophobic H-pocket residues of MRP1 and their corresponding residues in MRP2 are depicted in gray. Conserved residues are written in black, differing residues are written in red. Black dotted lines indicate hydrogen bonding. Unresolved hydrophobic tail of LTC4 is depicted in light gray.

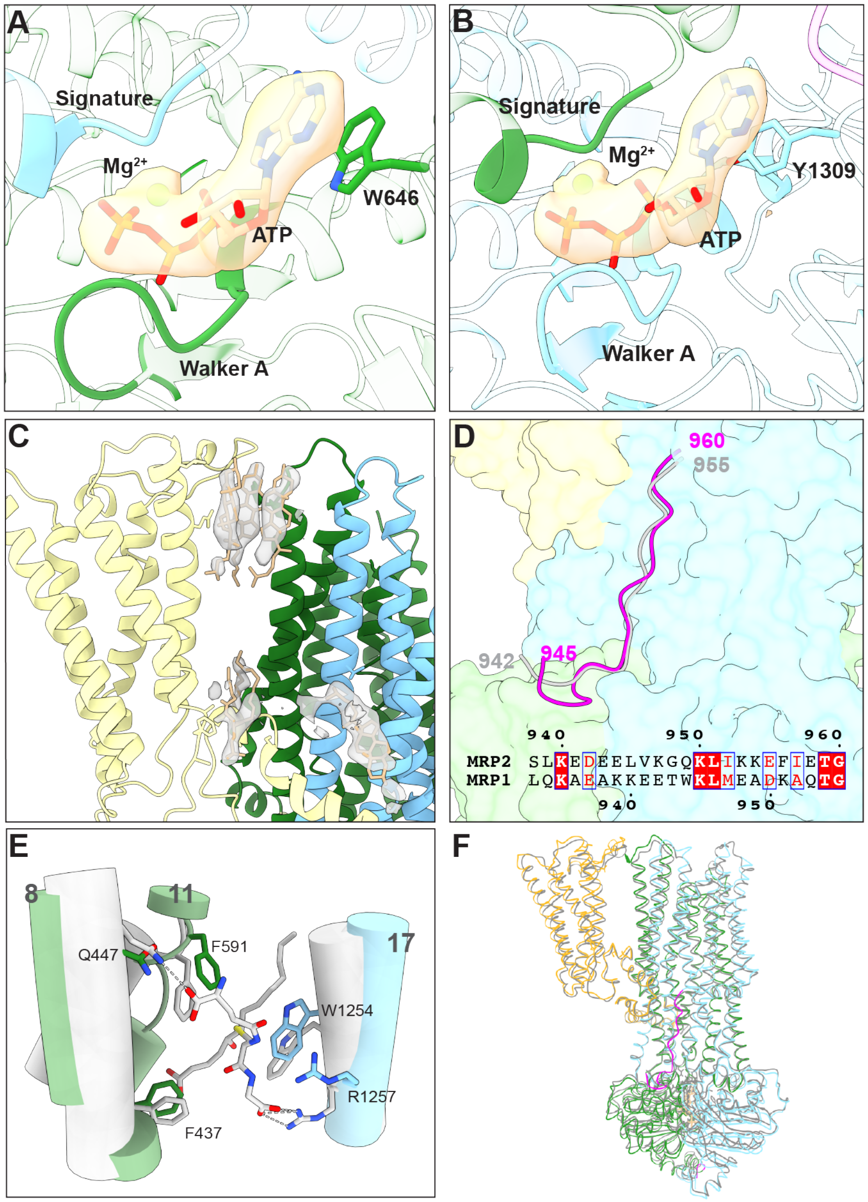

Supplementary figure 6. The structure of MRP2(E1462Q) in the ATP-bound, NBD-dimerized conformation

(A) Structure of the degenerate ATPase site. Residues from NBD1 are depicted in green, residues from NBD2 are depicted in blue. Cryo-EM density at contour level 0.109 corresponding to ATP and magnesium are depicted as tan surface.

(B) Structure of the consensus ATPase site, colored as in (A) with cryo-EM density of ATP and magnesium at contour level 0.109.

(C) Zoomed-in view of the TMD0 region, with cryo-EM density at contour level 0.0743 corresponding to cholesterol and lipids depicted as surface.

(D) Structural comparison of the MRP2 R domain (magenta) and MRP1 linker domain (gray), along with amino acid sequence alignment.

(E) Conformational changes at the substrate-binding site. The LTC4-bound structure is depicted in gray and the outward-facing structure depicted in green (TMD1) and blue (TMD2).

(F) Structural superposition of the MRP2(E1462Q) ATP-bound, pre-hydrolysis conformation (colored) with that of the WT MRP2 in the ATP/ADP-bound, post-hydrolysis conformation (PDB: 8JXU)

Supplementary Table 1. Data collection, processing, and structure refinement

	MRP2(E1462Q) inward-facing (EMDB-44833) (PDB 9BR2)	MRP2(E1462Q) Outward-facing (EMDB-44911) (PDB 9BUK)	MRP2/LTC4 (EMDB-45099) (PDB 9C12)	MRP2 Apo (EMDB-45159) (PDB 9C2I)
**Data collection and processing**				
Magnification	105,000	105,000	165,000	105,000
Voltage (kV)	300	300	300	300
Electron exposure ($e^{–}/Å^2$)	65	65	65	65
Defocus range (μm)	0.8 to 2.5	0.8 to 2.5	0.8 to 1.6	0.8 to 2.5
Pixel size (Å)	0.676	0.676	0.743	0.676
Symmetry imposed	C1	C1	C1	C1
Initial particle images (no.)	1,552,483	1,552,483	1,910,263	1,593,161
Final particle images (no.)	42,904	28,003	83,725	113,359
Map resolution (Å)	3.41	3.40	2.75	3.62
FSC threshold	0.143	0.143	0.143	0.143
**Refinement**				
Model resolution (Å)	3.4	3.4	2.9	3.6
FSC threshold	0.143	0.143	0.143	0.143
Model resolution range (Å)	3.2 to 3.8	4.3 to 3.7	2.7 to 3.7	3.5 to 4.0
Map sharpening *B* factor ($Å^2$)	70.7	62.7	58.4	116.4
Model composition				
Non-hydrogen atoms	11493	10815	10983	11186
Protein residues	1469	1394	1398	1422
Ligands	Magnesium: 1<br>ATP: 2<br>CLR: 5<br>UNL: 8	Magnesium: 2<br>ATP: 2<br>CLR: 5<br>UNL: 4	LTC4: 1<br>CLR: 5<br>UNL: 13	CLR: 5<br>UNL: 8
B factors ($Å^2$)				
Protein	66.40	56.86	104.86	65.58
Ligand	102.58	86.01	95.26	59.61
R.m.s. deviations				
Bond lengths (Å)	0.009 (0)	0.007 (1)	0.012 (4)	0.009 (0)
Bond angles (°)	1.083 (18)	0.937 (10)	1.745 (59)	1.060 (13)
Validation				
MolProbity score	1.53	1.41	0.99	1.57
Clashscore	3.78	3.15	2.16	4.24
Poor rotamers (%)	0	0	0	0
Ramachandran plot				
Favored (%)	94.67%	95.59%	98.13%	94.77%
Allowed (%)	5.33%	4.41%	1.87%	5.23%
Disallowed (%)	0	0	0	0

Supplementary Information References

1. Chen, S. *et al.* High-resolution noise substitution to measure overfitting and validate resolution in 3D structure determination by single particle electron cryomicroscopy. *Ultramicroscopy* **135**, 24–35 (2013).
